# Hyperthyroidism in a Twin Pregnancy With a Hydatidiform Mole and a Coexisting Live Fetus: Management Dilemmas

**DOI:** 10.1210/jcemcr/luaf013

**Published:** 2025-01-24

**Authors:** Samantha Jacobson, Jonathan-Raphaël Stetco, Richard Brown, Natasha Garfield

**Affiliations:** Faculty of Medicine, University of Sherbrooke, Sherbrooke, QC, Canada J1N 3C6; Faculty of Medicine, University of Sherbrooke, Sherbrooke, QC, Canada J1N 3C6; Department of Obstetrics and Gynecology, McGill University Health Center, Montréal, QC, Canada H4A 3J1; Division of Endocrinology, McGill University Health Centre, Montréal, QC, Canada H4A 3J1

**Keywords:** hyperthyroidism, hydatidiform mole, living fetus, twin pregnancy

## Abstract

Hyperthyroidism in twin pregnancies involving a hydatidiform mole and a coexisting live fetus is a rare condition requiring careful management. We present a 34-year-old pregnant woman at 12 weeks' gestation with severe nausea, vomiting, and mild vaginal bleeding. A transvaginal ultrasound revealed a dichorionic diamniotic twin pregnancy with 1 normal fetus and 1 hydatidiform mole, leading to hyperthyroidism from elevated β human chorionic gonadotropin levels. Conservative management without antithyroid medications, combined with regular monitoring, allowed the pregnancy to continue to term, resulting in the delivery of a healthy baby at 39 weeks. Postpartum management required treatment of retained products of conception. This case highlights the complexities in the management of complications for both mother and fetus.

## Introduction

Twin pregnancies involving a hydatidiform mole and a coexisting live fetus represent a rare form of gestational trophoblastic disease (GTD), occurring in approximately 1 in 22 000 to 100 000 pregnancies [1]. This condition poses significant management challenges, requiring enhanced monitoring and balancing treatment benefits against fetal risks. The simultaneous presence of a viable fetus and molar tissue, which is characterized by abnormal trophoblastic proliferation, can lead to severe complications, including hyperemesis gravidarum, preeclampsia, hyperthyroidism, and the potential for malignant transformation [2]. Fetal complications include abortion, growth restriction, and preterm labor [3]. Monitoring the fetus and deciding about pregnancy continuation, particularly in cases with metastasis, are critical.

Hyperthyroidism can be a life-threatening complication associated with molar pregnancies as it can progress to severe thyrotoxicosis and even thyroid storm [4]. It arises due to the excessive production of β human chorionic gonadotropin (β- hCG) by the molar tissue, which can mimic TSH and overstimulate the thyroid gland [4]. This leads to elevated thyroid hormones levels, with clinical manifestation such as tachycardia, weight loss, and persistent nausea [5].

Effective management of hyperthyroidism in a twin molar pregnancy involves a multidisciplinary approach, including obstetricians, endocrinologists, and oncologists. We present a 34-year-old pregnant woman with a hydatidiform mole and a coexisting live fetus with comorbid hyperthyroidism.

## Case Presentation

A 34-year-old gravida 2, para 1 female at 12 weeks' gestation presented with severe nausea, vomiting, and mild vaginal bleeding. She had 1 prior uncomplicated full-term vaginal delivery 14 years earlier; the baby was delivered at 39 weeks at 2.7 kg (5.95 pounds). She had no significant medical history. Her prepregnancy weight was 63.6 kg (140.2 pounds, body mass index 20.1). At 12 weeks, the patient experienced nausea, exertional dyspnea, and a weight loss of 1.4 kg (3 pounds).

## Diagnostic Assessment

**Figure 1.**
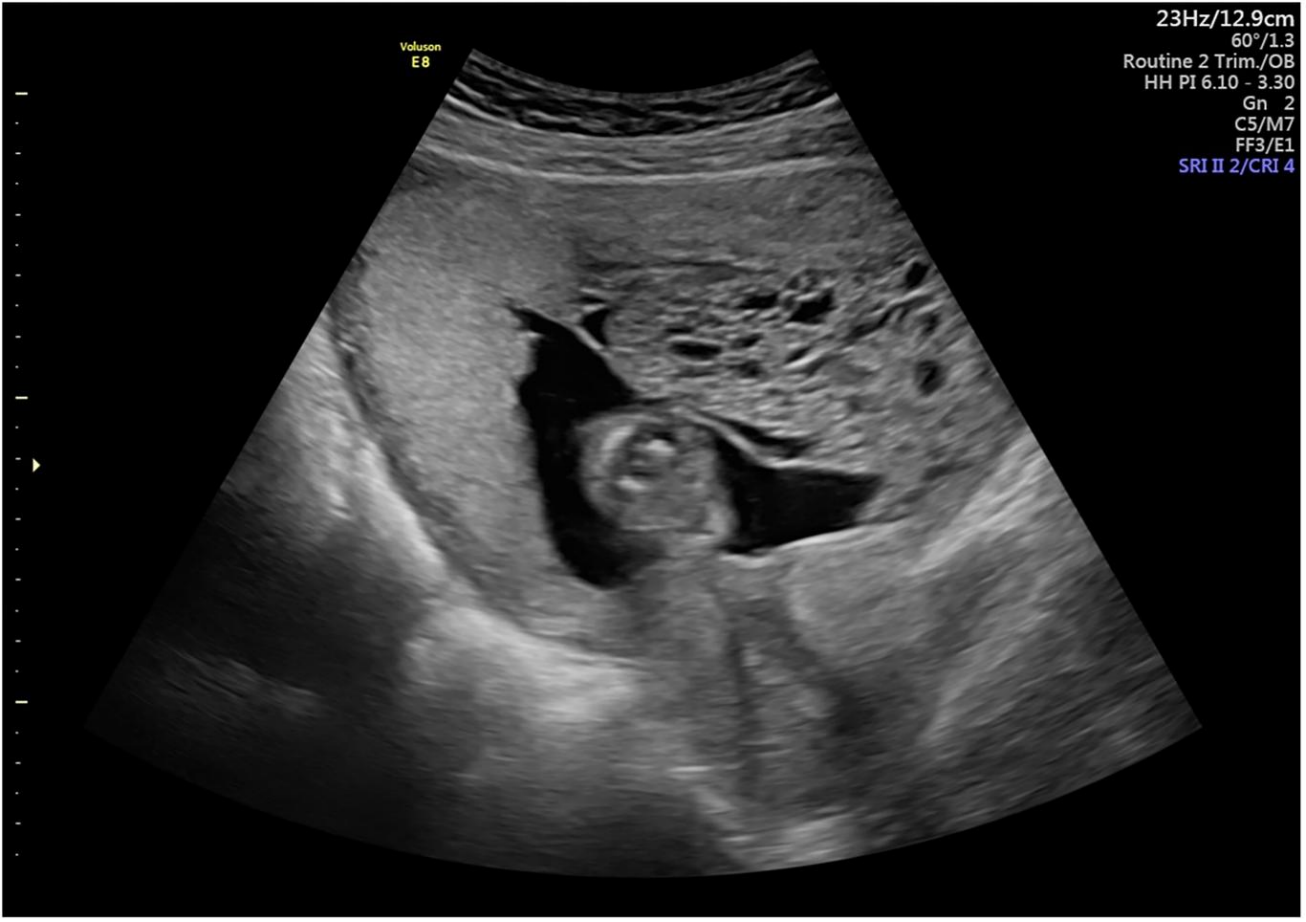
The normal placenta is shown on the left, while the molar placenta is on the right, with the gestational sac and fetus centrally positioned.

Physical examination revealed a blood pressure of 117/79 mmHg, heart rate of 78 bpm, mild lid lag, fine resting tremor, and a palpable thyroid without enlargement or distinct nodules. A transabdominal ultrasound at 12 weeks' gestation revealed a dichorionic diamniotic twin pregnancy, with 1 normal fetus and 1 hydatidiform mole characterized by a large cystic area measuring 70 × 50 × 80 mm adjacent to the normal placenta (Fig. 1). Fetal heart rate was 159 bpm; amniotic fluid volume was normal. Laboratory results showed TSH <0.02 mIU/L (1.02 pM) (reference range: 0.4-4.0 mIU/L; 1-15 pM), free T4 24 pmol/L (1.02 pM) (reference range: 10-25 pmol/L; 0.8-2.0 ng/dL), free T3 9.79 pmol/L (0.64 ng/dL) (reference range: 3-8 pmol/L; 0.2-0.5 ng/dL), and β-hCG 379 393 mIU/L (379.39 IU/L) (reference range: 27 107-201 165 mIU/mL; 27.1-201.2 IU/L). TSH (hypersensitive) and free T4 were measured using a chemiluminescent immunoassay. Antithyroid peroxidase antibodies and TSH-receptor antibodies were negative. A chest X-ray ruled out metastases. The patient was diagnosed with a complete molar pregnancy coexisting with a viable twin and associated hyperthyroidism.

## Treatment

The patient was managed conservatively without any antithyroid medications, as her free T4 and free T3 normalized and symptoms were mild. She was started on aspirin 160 mg daily for prevention of gestational hypertensive disorders.

## Outcome and Follow-up

**Figure 2.**
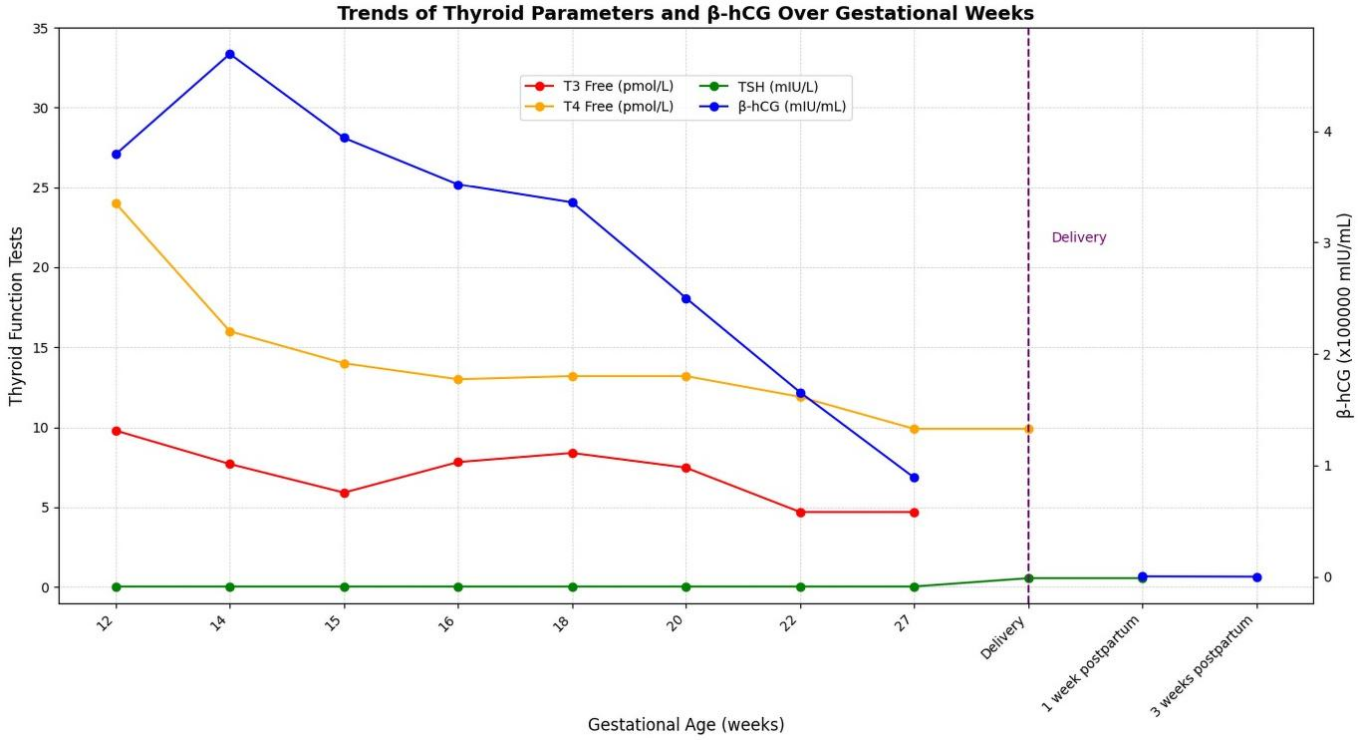
Thyroid function and β human chorionic gonadotropin levels over time in a patient with molar pregnancy and coexisting live fetus.

By 16 weeks' gestation, her weight was 63.3 kg (139.5 pounds), up 1.1 kg (2.4 pounds), and by 24 weeks, it was 70.2 kg (155 pounds) due to a nutritional supplement (Boost®) and a high-protein diet that were introduced. Transabdominal ultrasound at 27 weeks showed a fetal heart rate of 140 bpm and normal amniotic fluid volume with a maximum vertical pocket of 4.3 cm (reference range: 2-8 cm); the molar area measured 104 × 25 × 90 mm. Biweekly lab tests for β-hCG and thyroid functions (Fig. 2) showed a downtrend in β-hCG levels, while TSH remained less than 0.02 mIU/L (1.02 pM) (reference range: 0.4-4.0 mIU/L; 1-15 pM). Free T3 and T4 levels remained within the normal ranges. She was followed by a multidisciplinary team including, maternal-fetal medicine, endocrinology, and psychiatry, for support. At 36 weeks, her weight reached 82.4 kg (181.5 pounds). Transabdominal ultrasound at 37 weeks showed a fetal heart rate of 150 bpm; amniotic fluid volume was normal with a maximum vertical pocket of 3.8 cm (reference range: 2-8 cm) and an amniotic fluid index of 11.8 cm (reference range: 8.2-14 cm). Fetal thyroid appeared normal. Molar area at the time was 92 × 20 × 87 mm. Growth velocity was maintained throughout pregnancy.

The patient delivered a healthy baby girl at 39 1/7 weeks gestation. The baby weighed 3160 grams (6 pounds and 15 ounces) and had Apgar scores of 9 at 1 minute and 9 at 5 minutes. The delivery was complicated by shoulder dystocia but there were no consequences to the baby. The patient experienced persistent vaginal bleeding postdelivery, and an ultrasound revealed retained products of conception (RPOC). A dilation and curettage were performed 2 days postdelivery, after a trial of Cytotec failed to expel the RPOC.

Histopathology of the RPOC confirmed a molar pregnancy and the presence of a hydatidiform mole. The following 2 consecutive postpartum β-hCG tests were 265 mIU/L (0.265 IU/L) (reference range: 10–10 000 mIU/L; 0.1-10 IU/L) 1 week postpartum and 3.3 mIU/L (0.0033 IU/L) (reference range: 0-5000 mIU/L; 0-5 IU/L) 3 weeks postpartum.

## Discussion

Managing a twin pregnancy complicated by a hydatidiform mole and associated hyperthyroidism presents a unique set of challenges due to the rarity and complexity of the condition. Hyperthyroidism in GTD arises from β- hCG secretion from trophoblastic tissue, which stimulates thyroid hormones through molecular mimicry [6]. This occurs because the α subunit of β-hCG is structurally similar to the α subunit of TSH [6]. This structural similarity allows β- hCG to interact with TSH receptors on the thyroid gland, albeit with significantly lower potency—approximately 4000 times less than TSH [5]. This increase in β-hCG can therefore lead to hyperthyroidism, especially in states of supraphysiologic β-hCG levels such as is seen in GTD [6]. Clinical differentiation of hyperthyroidism from normal pregnancy is challenging due to overlapping symptoms, such as tachycardia, dyspnea, gastrointestinal symptoms, and changes in bowel movements. Failure to gain adequate weight or unexplained weight loss may suggest hyperthyroidism [7]. While mild tachycardia is physiological during pregnancy, excessively elevated heart rates should raise suspicion of thyroid dysfunction [7]. Additionally, tremors and persistent nausea and vomiting extending beyond the first trimester also warrant consideration [7].

Early detection and management of GTD through first-trimester ultrasonography and serum β-hCG testing have reduced the incidence of late-presentation hyperthyroidism [4]. The treatment of hyperthyroidism in GTD is similar to the management of hyperthyroidism resulting from primary thyroid conditions [4]. Primary treatment involves the administration of antithyroid drugs (ATDs) to control the hyperthyroid state [8]. Uncontrolled maternal hyperthyroidism is linked to higher rates of miscarriage and preterm delivery [3]. Miscarriage, which occurs in 15-25% of all pregnancies, is significantly more common in women with thyroid dysfunction, with odds ratios ranging from 3.4 to 9.56 depending on maternal TSH levels and antibody status [9]. Drugs such as propylthiouracil (PTU) or methimazole (MMI) are used to inhibit thyroid hormone synthesis. PTU is preferred in the first trimester to minimize risks of congenital malformations. After this trimester, MMI is recommended to reduce PTU-induced liver failure risks [8]. However, these drugs can cross the placenta and cause fetal hypothyroidism and goiter [3]. While the fetal thyroid becomes functional around the 11th–12th week of gestation [10], maternal β-hCG levels peak during the first trimester. This peak could theoretically stimulate the fetal thyroid gland, especially if maternal β-hCG levels are abnormally elevated (eg, in GTD or hyperemesis gravidarum) [3]. The elevated levels of β-hCG seen in GTD stimulate the maternal thyroid gland and thus result in elevated free T4 and free T3 levels that could cross the placenta and expose the fetus to increased levels of thyroid hormones. However, this effect is typically minimal as the placenta also regulates maternal thyroid hormone transport and shields the fetus to some extent. In conditions like Graves' disease, the additional crossing of thyroid-stimulating antibodies can cross the placenta and directly stimulate the fetal thyroid, increasing the risk of fetal hyperthyroidism [3]. The overall risk of fetal hyperthyroidism remains low, with approximately 1% of children born to mothers with Graves' disease experiencing this condition [11].

To balance maternal-fetal risks, the lowest effective dose of ATDs is used to maintain maternal free T4 levels in the high normal range. Overtreatment risks fetal hypothyroidism and goiter, detected on fetal ultrasound through findings such as a goiter, growth restriction, and delayed bone age as well as cause-sustained fetal heart rates exceeding 160 bpm [12, 13]. Adjusting or discontinuing ATDs typically normalizes thyroid function and reduces goiter size [12]. Development of a fetal goiter can introduce complications during pregnancy and delivery, including polyhydramnios caused by impaired fetal swallowing, cervical dystocia during labor, and obstruction of the fetal airway postpartum [12, 13]. Rarely, fetal thyrotoxicosis may lead to severe outcomes such as heart failure, fetal hydrops, or intrauterine death [14]. Regular thyroid function tests, conducted every 2 to 4 weeks, ensure the lowest dose of ATDs is used to minimize fetal exposure [7]. β-blockers, like propranolol, are also administered to manage symptoms such as tachycardia and tremors by blocking the peripheral effects of excess thyroid hormones [3]. Although propranolol is considered safe with no teratogenic effects, its prolonged use during pregnancy should be avoided due to reports linking chronic administration with intrauterine growth restriction [7]. Alternative treatments include thyroidectomy, which, if needed, is safest in the second trimester. It is reserved for severe or unresponsive cases [3, 7]. In severe cases of hyperthyroidism or when there is a need for urgent surgical intervention, therapeutic plasmapheresis can be considered [4]. Plasmapheresis rapidly reduces circulating thyroid hormone levels by removing the hormone bound to serum proteins, which is critical in stabilizing the patient before surgery [4].

Definitive hyperthyroidism treatment in molar pregnancy is the surgical evacuation of the molar tissue [4]. This procedure leads to a rapid decline in β-hCG levels, which in turn normalizes thyroid function. In many cases, patients become euthyroid and do not require further ATDs after the evacuation. In most cases, β-hCG levels follow a downward trend after treatment, as observed in this patient, with approximately 80% to 90% of complete molar pregnancies resolving without further intervention [15]. However, β-hCG trajectories can vary; a plateau in β-hCG levels may indicate residual trophoblastic tissue, while persistently rising β-hCG levels, observed in approximately 15% to 20% of cases, are suggestive of progression to GTN [15]. If there are signs of progression, such as rising β-hCG levels or worsening symptoms, prompt surgical evacuation of the molar tissue is necessary to prevent complications and achieve definitive treatment. In this case, the mole was not removed due to the presence of a viable twin. Keeping the hydatidiform mole increases the risk of severe thyrotoxicosis and even thyroid storm due to the high levels of β-hCG produced, which can overstimulate the thyroid [16]. Additionally, 15% to 20% of hydatidiform moles develop into GTN, a malignant transformation [17]. Regular ultrasounds are essential to monitor the growth of the normal fetus, the size of the molar pregnancy, and any complications such as theca lutein cysts or excessive amniotic fluid [18]. Decision-making about continuing the pregnancy depends on fetal viability, the absence of severe complications like preeclampsia or hyperthyroidism, and the ability to provide comprehensive monitoring [17]. If there is evidence of metastasis, fetal compromise, or severe maternal health risks, termination of the pregnancy is often recommended [17]. Postevacuation management includes chemotherapy for GTN, along with regular β-hCG surveillance to ensure no recurrence [18].

In the case of our patient, hyperthyroidism was managed conservatively due to the patient's mild symptoms. Her lack of significant medical history, including the absence of cardiac disease, supported the decision for conservative management. Healthy patients without significant cardiac disease can withstand conservative management of mild hyperthyroidism. Additionally, the decision for conservative management was supported by the improving levels of free T4 and free T3, along with a corresponding decline in the serum β-hCG levels. A multidisciplinary team, including obstetricians, endocrinologists, and, in this case, a psychiatrist, played a crucial role in the management of the patient, providing continuous monitoring and tailored therapeutic strategies to address both maternal and fetal health. Extensive counseling was provided to the patient and her partner, discussing potential complications and management options.

## Learning Points

- Twin pregnancies involving a hydatidiform mole and a coexisting live fetus are rare and present management challenges, particularly due to potential complications such as hyperthyroidism and the risk of GTN.
- Hyperthyroidism in molar pregnancies arises due to excessive β-hCG production by the molar tissue, which can mimic TSH and overstimulate the thyroid gland, necessitating close monitoring of thyroid function and β-hCG levels throughout the pregnancy.
- Conservative management can be a viable option in cases where hyperthyroidism symptoms are mild, involving regular monitoring without the use of antithyroid medications, as demonstrated by the successful continuation of the pregnancy to term in this case.
- Postpartum monitoring is crucial for detecting and managing retained RPOC and ensuring no progression to GTN, highlighting the need for diligent follow-up even after a successful delivery.

## Contributors

All authors made individual contributions to authorship. S.J. was involved in the writing, submission, and preparation of tables. J.S. was involved in writing the article. R.B. and N.G. were involved in the diagnosis and management of this patient. All authors reviewed and approved the final draft.

## Data Availability

Data sharing is not applicable to this article as no datasets were generated or analyzed during the current study.
